# Operationalizing Team Science at the Academic Cancer Center Network to Unveil the Structure and Function of the Gut Microbiome

**DOI:** 10.3390/jcm14062040

**Published:** 2025-03-17

**Authors:** Kevin J. McDonnell

**Affiliations:** Center for Precision Medicine, Department of Medical Oncology & Therapeutics Research, City of Hope Comprehensive Cancer Center, Duarte, CA 91010, USA; kemcdonnell@coh.org

**Keywords:** City of Hope, microbiome, systems biology, artificial intelligence

## Abstract

Oncologists increasingly recognize the microbiome as an important facilitator of health as well as a contributor to disease, including, specifically, cancer. Our knowledge of the etiologies, mechanisms, and modulation of microbiome states that ameliorate or promote cancer continues to evolve. The progressive refinement and adoption of “omic” technologies (genomics, transcriptomics, proteomics, and metabolomics) and utilization of advanced computational methods accelerate this evolution. The academic cancer center network, with its immediate access to extensive, multidisciplinary expertise and scientific resources, has the potential to catalyze microbiome research. Here, we review our current understanding of the role of the gut microbiome in cancer prevention, predisposition, and response to therapy. We underscore the promise of operationalizing the academic cancer center network to uncover the structure and function of the gut microbiome; we highlight the unique microbiome-related expert resources available at the City of Hope of Comprehensive Cancer Center as an example of the potential of team science to achieve novel scientific and clinical discovery.

## 1. Introduction

We exist in a state of mutualism with an array of microorganisms that have co-evolved with us to thrive within specific biological niches throughout the human body [1,2,3]. The gut colonic niche stands apart as the largest and most diverse reservoir of microbiota in the human body [4,5]. To the degree that the microbiome influences our health and disease predisposition, arguably, the gut microbiome exerts the greatest influence [6,7,8]; these effects may include cancer prevention and predisposition, as well as the modulation of the cancer therapeutic response [9,10]. Therefore, attaining a better understanding of gut microbiome composition and associated biological effects may reveal new approaches to mitigating risk and improving clinical outcomes [11,12].

Innovations in technology and basic science research have made possible a rapid expansion of our knowledge of the gut microbiome. Emerging capabilities in the computational sciences, specifically systems biology and artificial intelligence, promise continued mechanistic and therapeutic discoveries. Academic clinical cancer networks, with their care of diverse cancer populations, access to advanced, core experimental facilities, and concentration of pioneering scientific experts, serve as natural accelerators and efficient incubators of microbiome research. In this review, we examine current knowledge of the gut microbiome, explore recent innovations in microbiome research, and, finally, focus on the facilitative role of the academic cancer center network in advancing microbiome investigation through a description of resources and research initiatives at the City of Hope (COH) Comprehensive Cancer Center.

## 2. States of the Gut Microbiome in Health and Disease

**Figure 1:**
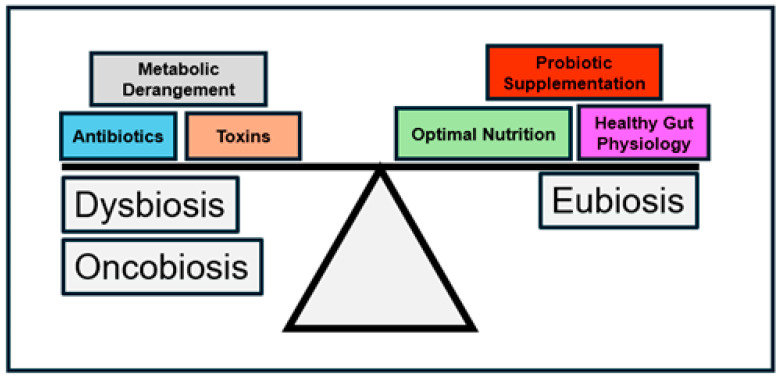
Numerous factors determine states of eubiosis, dysbiosis and oncobiosis. Many different variables determine whether the microbiome promotes health (eubiosis) or predisposes to disease (dysbiosis and oncobiosis). Both environmental and genetic factors contribute to the final state of microbiome health.

The human colon hosts a staggeringly large and diverse population of bacteria [13,14]. Our bodies provide these bacteria a stable, homeostatic environment that furnishes secure residence, a source of nutrients, opportunity for growth, and space for reproduction [15,16]. In return, from these bacteria, we derive essential cofactors, critical metabolites, and beneficial end products of digestion [11,17,18]. Moreover, these bacteria exert salutary immunological and genetic modulatory effects and inhibit the colonization of competing pathogenic bacteria [19,20,21]. The coevolution of the “seed” (gut bacteria) and “soil” (the human gut) of the gastrointestinal tract produces an optimized state of bacterial growth and mutual benefit: eubiosis (Figure 1).

Together, seed and soil conditions shape the microbial composition of eubiosis. The establishment of eubiosis begins at birth with the initial seeding of a sterile, abacterial gastrointestinal tract [22,23]. Depending on the mode of delivery at birth, vaginal versus cesarean section, the colon will acquire a microbiome predominated by bacteria of the Firmicutes and Bacteroidetes phyla (vaginal delivery) or Firmicutes and Actinomycetota phyla (cesarean section) [24,25]. Subsequent breast- or bottle-feeding results in the colonization by Bifidobacterium species with significant numbers of either Enterobacteriaceae (bottle-feeding) or Lactobacillus (breastfeeding) species [26,27]. By two years of age, however, these bacterial populations converge towards a mature, eubiotic bacterial population comprising predominantly species from the Bacteroidetes and Firmicutes phyla with Faecalibacterium Prausnitzil representing the most common species in the adult colon [28,29].

Eubiosis requires maintenance and remains vulnerable to disruption—compromise of eubiosis produces dysbiosis. Numerous factors contribute to dysbiosis; exposure to toxic agents, antibiotics, and nocuous dietary components may eradicate native, beneficial bacterial populations allowing pathogenic bacteria to proliferate in their place [30,31,32]. For example, prolonged or multiple episodes of antibiotic use diminish Bacteroidetes and Firmicutes populations, allowing dysbiotic populations, e.g., Clostridium Difficile, to proliferate, potentially resulting in serious gut pathology [33,34,35]; restoration of eubiotic bacterial populations may necessitate active intervention [36,37]. Dysbiosis, if extreme and not corrected, produces severe health sequelae.

## 3. The Impact of Gut Microbiome Dysbiosis on Cancer Predisposition, Treatment Toxicity, and Therapeutic Efficacy

Extreme shifts in the gut microbiome population cause dysbiosis and, potentially, oncobiosis; investigations associate gut oncobiosis with increased cancer predisposition, more frequent and severe drug side effects, and attenuated clinical effectiveness of treatment [38,39,40,41,42].

The microbiome alters mechanisms of cancer initiation and progression through the modulation of several fundamental gut-associated activities such as inflammatory control, immune function, maintenance of genomic integrity, and epigenetic regulation [43,44]. Key bacterial populations play critical roles in accomplishing these activities and influencing cancer predisposition. Previous studies have identified specific colon cancer-promoting Escherichia coli, Bacteroides fragilis, and Fusobacterium nucleatum bacteria [45,46,47].

Gut microbiome production of genotoxins may significantly contribute to increased cancer predisposition. Genotoxic agents induce DNA damage, a prelude to cancer initiation and a driver of cancer progression. The gut microbiome produces various genotoxins that include colibactin, indolimines, and cytolethal distending toxins (CDTs).

Patients with inflammatory bowel disease (IBD) frequently demonstrate microbiome shifts characterized by increased Escherichia coli populations [48,49]. Certain strains of Escherichia contain a biosynthetic gene cluster that allows the synthesis of colibactin, a polyketide peptide [50]. The colibactin peptide promotes the formation of DNA interstrand crosslinks within colonic epithelial tissue, causing genomic instability and initiating neoplastic transformation [51,52]. Another group of colonic genotoxins, the indolimines, produced predominantly by the gut microbe, Morganella morganii, also predisposes the gut epithelium to neoplastic transformation. Investigations demonstrate that indolimines produce DNA double-strand breaks that induce oncogenic nucleic acid damage and initiate cancer. Similarly, CDTs, a class of bacterial proteins, also produce DNA double-strand breaks. CDTs exert other cancer-inducing effects beyond DNA double-strand break induction. Notably, CDTs arrest the cell cycle, leading to cellular architectural distortion and pathophysiologic dysfunction. A host of gut bacteria produce CDTs: Salmonella, Campylobacter, Helicobacter, and Shigella species, among others.

Gut microbiome-induced cancer predisposition extends beyond the proximal colon epithelium. Gut microbiome dysbiosis predisposes to breast cancer; specific gut bacteria may increase circulating estrogen levels, increasing the risk for mammary neoplastic transformation [53,54,55]. Similarly, gut bacteria may affect the production of androgens, modifying the risk for the development of prostate cancer [56,57]. Finally, the gut microbiome, by influencing the bodily inflammatory state, alters susceptibility to lung cancer [58,59,60].

In addition to cancer predisposition, gut microbiome dysbiosis may alter the response to cancer therapy. This altered response manifests both as worsened drug side effects and diminished therapeutic efficacy [42,61,62,63]. The gut microbiome plays a critical role in the metabolism of many chemotherapeutic agents [64,65]. Gut microbiome metabolic detoxication may help attenuate gastrointestinal distress, such as diarrhea, intestinal mucositis, and nausea, which frequently accompanies the administration of chemotherapeutic agents [66,67,68,69,70]; however, the gut microbiome may also exacerbate such side effects [71,72]. Normally, the liver detoxifies the chemotherapeutic irinotecan through glucuronidation. Beta-glucuronidase produced by dysbiotic gut bacteria may reverse glucuronidation and detoxification, leading to a reactivation of irinotecan that directly damages the gut epithelium and increases chemotherapy-associated gastrointestinal dysfunction [73,74]. Gut dysbiosis may also worsen side effects resulting from bone marrow transplant (BMT) to treat hematologic cancer. Graft-Versus-Host-Disease (GVHD), a potentially life-threatening side effect of BMT, is more likely to occur post-transplant in association with dysbiotic gut microbiomes with increased concentrations of Bacteroides, Prevotella, and Enterococcus, and decreased concentrations of Lactobacillus, Clostridia, and Akkermansia bacterial populations [75,76,77]. Such dysbiosis may compromise the intestinal barrier, resulting in a worsening of the body’s inflammatory state and increasing the risk of GVHD [77,78]. In addition to exacerbating the side effects of cancer treatment, gut dysbiosis may diminish the cancer therapeutic response [79,80]. This therapeutic diminishment affects traditional cytotoxic chemotherapy as well as precision, molecularly targeted agents and immunotherapies [81,82,83].

The gut microbiota metabolizes cytotoxic chemotherapeutic drugs, producing both beneficial and harmful end effects [84]. The gut bacterium Raoultella planticola deglycosylates doxorubicin; deglycosylated doxorubicin produces less epithelial damage than glycosylated doxorubicin and substantively reduces toxic side effects [85,86]. However, other bacteria, notably Klebsiella pneumonia and Escherichia coli, may excessively metabolize and degrade doxorubicin, thereby reducing clinical efficacy [87,88]. In addition to modulating cytotoxic chemotherapeutic efficacy, the gut microbiome may alter the effectiveness of precision therapeutics [89,90]. Oncologists utilize two classes of precision drugs: monoclonal antibodies and small molecule inhibitors. The gut microbiome regulates the biological activities of both classes. Studies have discovered that the gut microbiota impacts the efficacy of the monoclonal antibodies trastuzumab and cetuximab [91,92,93]; gut microbiota also affect clinical outcomes in patients treated with the small molecule inhibitors lapatinib and imatinib [94,95].

The gut microbiome, as well, affects the efficacy of immune treatments such as immune checkpoint inhibitors (ICIs) and Chimeric Antigen Receptor T-cell (CAR-T) therapy. Patients with significant Bifidobacterium longum, Prevotella copri, and Faecalibacterium prausnitzii populations demonstrate improved clinical responses to ICIs [94,96,97]; in contrast, patients with higher proportions of Bacteroides, Ruminococcus obeum, and Roseburia intestinalis have decreased responses [98,99]. Gut microbiome populations influence the response to CAR-T therapy. Individuals with gut microbiomes enriched with Bacteroides, Ruminococcus, Faecalibacterium, Akkermansia, and Eubacterium species respond better to CAR-T therapy [100,101]. However, those patients with gut microbiomes dominated by other microbial species, especially Veillonellaceae, demonstrated incomplete responses [102,103]. Furthermore, complications of CAR-T, specifically cytokine release syndrome (CRS), occur more frequently and significantly in patients with gut microbiomes with higher proportions of Bifidobacterium, Leuconostoc, Stenotrophomonas, and Staphylococcus species; oppositely, some microbial species, e.g., Collinsella, may lessen the risk of cytokine-related complications [104,105].

**Figure 2:**
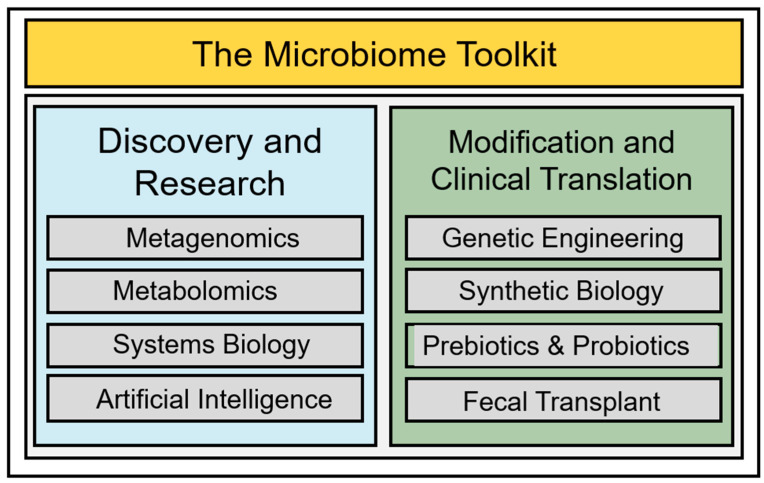
Multiple Tools are Available to Microbiome Investigators and Clinicians. Researchers and physicians have several methods and protocols available to them to advance microbiome discovery and clinical translation.

Deciphering compositional differences among eubiotic, dysbiotic, and oncobiotic gut conditions allows investigators to make important associative connections between bacterial populations and disease states; further, the discovery of functional, mechanistic differences permits scientists to move beyond mere description and advance toward the development and implementation of active preventative and therapeutic interventions [106,107]. Recently, innovations in next-generation diagnostics, molecular cell biology, and computational sciences have paved tractable pathways to realize these advances and provided a toolkit for advancing research and clinical translation (Figure 2). Among the many innovations in this toolkit, new methods in metagenomics, mass spectrometry, systems biology, and artificial intelligence have uncovered the molecular bases of gut eubiosis, dysbiosis, and oncobiosis [108,109,110,111].

## 4. Towards a More Complete Accounting of the Gut Microbiome: Genomic and Functional Advances

### 4.1. The Promise of Metagenomics

Metagenomics provides a comprehensive molecular genetic description of the gut microbiome through genomic sequencing of the bacterial consortium en masse [112,113]. Downstream computational deconvolution identifies, distills, and molecularly clarifies individual consortium members [114]. Metagenomics demonstrates great scientific utility, overcoming several limitations of precedent bacterial genomic sequencing methods. Notably, laboratory-cultured gut microbes, frequently fastidious and typically the genome source for sequencing microbiome constituents, limit the ability to perform a complete genomic assessment. Metagenomic approaches, because they directly sequence a biological sample without the requirement for intermediary laboratory culturing, enable a complete ensemble, highly precise, and exceptionally detailed genomic assessment of the microbiome. Further advances in gut microbiome metagenomics, such as genome-resolved methods and high-throughput sequencing, promise to further accelerate the molecular accounting of complex gut bacterial populations [115,116]. Insights from metagenomic analyses antecede downstream functional assessments and predicate developments in clinical intervention.

### 4.2. Deciphering the Molecular Metabolome of the Gut Microbiome

Bioactive molecules and metabolites generated by the gut microbiome influence cancer predisposition and modulate cancer treatment effects [14,40,42,65,117,118,119,120]. Advances in molecular diagnostics, specifically innovations in mass spectrometry, significantly enhance our ability to discover and detect these bioactive molecules and understand their physiologic significance [121,122,123].

Mass spectrometry quantitates the mass to electronic charge ratio (m/z) of ions generated from a molecular substance [124,125]. Molecules have specific, identifying m/z signatures reflecting each substance’s unique isotopic and chemical composition; mass spectrometry detects, molecularly deconstructs, and precisely quantifies both previously well-characterized substances and unknown, novel chemicals [126,127]. Mass spectrometry performs reliably and versatilely; mass spectrometry ably analyzes the composition of highly diverse and heterogeneous materials [128,129]. These broad analytic capabilities well qualify mass spectrometry to appraise the expansive spectrum of gut microbiome bioactive molecules.

In particular, mass spectrometric characterization provides detailed molecular accounting of the gut bacterial metabolome [130,131]. Metabolic products of gut bacteria include peptides, lipids, fatty acids, carbohydrates, and nucleic acids [132,133]. These small molecules mediate critical biological activities and significantly influence gut physiology. Mass spectrometric cataloging of these metabolites contributes to a mechanistic understanding of both healthy and pathogenic states of the gut microbiome; recent advances have accelerated these understandings.

Recent notable advances in mass spectrometry include innovations in technology and computation. Two related, novel technological innovations, single-cell mass spectroscopy [134,135,136] and spatially resolved mass spectroscopy [137,138,139], unveil metabolic processes occurring within specific regions and among heterogeneous constituents of the gut microbiome. Such levels of resolution and specificity facilitate the development of complex, highly detailed mechanistic models of the interactions among the many gut microbial species and between the microbiome and its host. The application of a systems biological framework to these interactions confers an intuitive structure upon these complex interactions [140,141,142]. The creation of such highly detailed and precise mechanistic models, however, requires tremendous analytic expertise and significant computational resources. Innovative computational approaches provide solutions to overcome the logistical and capital impediments to continued metabolomic discovery.

The considerable computational challenges of identifying, deconvoluting, and deciphering the metabolic mechanisms of the gut microbiome arise simply from the sheer volume of both previously known metabolites and unknown, uncharacterized metabolic products. To optimize the identification of previously annotated metabolites, computational scientists employ strategies such as community-enhanced mass spectrometric reference libraries [143,144] and high-efficiency molecular matching protocols [145,146]. In parallel, the utilization of highly efficient molecular structural modeling approaches allows for the rapid, proficient identification and cataloging of newly discovered microbiome metabolites [147,148]. Comprehensive microbiome metabolomic libraries serve as data compendia for subsequent analytics including structure–function prediction tools and systems biological workflows; these analytics enable scientists to develop deep, rigorous mechanistic descriptions of microbiome–host interactions [149,150,151].

Microbiome mechanistic descriptions serve as bases for the conception and invention of therapies to prevent and manage symptoms and treat diseases associated with gut dysbiosis. For example, mechanism-informed lifestyle interventions promote eubiosis; bioengineers leverage mechanistic insights to accelerate the design and utilization of clinical interventions such as prebiotic supplementation and fecal transplantation to mitigate oncology treatment-associated side effects and maximize therapeutic efficacy [152,153,154]. Developing a more complete understanding of the metabolic bioactive molecules produced by the gut microbiome will continue to catalyze mechanistic and therapeutic discovery [12,155,156].

### 4.3. Towards a More Complete Accounting of the Gut Microbiome Molecular Inventory

Mass spectrometry achieves a detailed accounting of the bioactive molecules produced by the gut microbiome. These bioactive molecules may cause direct genotoxic effects as well as modulate oncogenic pathways, predisposing patients to cancer development [157,158,159]. Modulatory bioactive molecules include short-chain fatty acids, polyphenols, bile acid metabolites, and tryptophan derivatives.

Fatty acids have linear hydrocarbon backbones terminating in an acidic carboxyl group [160,161]. Chemists classify fatty acids with a backbone of five or fewer carbon atoms as short-chain fatty acids (SCFAs) [162,163]. Physiologically, SCFAs mediate important biological functions, notably influencing cancer initiation, progression, and response to therapy [164,165,166]. The gut microbiome serves as a primary source of SCFAs, synthesized primarily through the fermentation of dietary fiber. Important SCFAs produced by the gut microbiome include acetate, propionate, and butyrate. Bacteroides and Faecalibacterium bacteria produce the greatest amounts of gut SCFAs [167,168].

Another class of gut bioactive products, the polyphenols, exert powerful, generally oncoprotective effects [169,170]. Chemically, polyphenols contain multiple hydroxylated aromatic rings [171,172]. The gut microbiome produces several important classes of polyphenols: hydrophenylpropionic acid, hydroxyphenylacetic acid, and the urolithins [173,174,175]. These gut polyphenols derive principally from bacterial metabolism of ingested parent, plant-based substances rich in polyphenol substrates [176,177]. Microbiome polyphenols inhibit oncogenesis by reducing colonic inflammation, mitigating oxidative processes, and inhibiting oncogenic molecular signaling pathways [178,179,180]. Lactobacillus, Bifidobacterium, Roseburia, Akkermansia, and Faecalibacterium microbial populations play central roles in the metabolism and processing of ingested polyphenols [181,182,183,184]. Disruptions of these microbial populations abrogate associated oncoprotective effects, potentially predisposing to cancer development.

The liver synthesizes and releases bioactive bile salts that serve primarily to facilitate fat digestion [185,186]. Bile acids, though, at excessive levels, may have harmful effects producing colonic inflammation, nucleic acid oxidation, and cell cycle disruption [187,188,189,190,191,192,193]. These pathophysiologic disruptions activate colon cancer initiation pathways and promote oncologic progression [194,195,196]. The gut microbiome, through the metabolic processing of gut bile acids, primarily cholic acid and chenodeoxycholic acids, may exacerbate these risks through the synthesis of secondary bioactive bile metabolites [197]. Secondary gut microbiome-generated metabolites, such as deoxycholic acid, lithocholic acid, and hydrogen sulfide, amplify the oncogenic effects of bile acids [198,199,200]. Investigators have implicated Clostridium Bacteroides and Eubacterium as the major gut bacteria responsible for the generation of harmful secondary bile acid products [201,202,203].

We routinely ingest tryptophan, an essential amino acid, as part of a complete, balanced diet. Tryptophan serves as an indispensable building block for protein synthesis, but the cell also utilizes tryptophan to construct myriad bioactive molecules [204,205,206]. Gut microbiota, notably Bacillus, Pseudomomas, and Bacteroides species, metabolize tryptophan to generate several biologically important substances, specifically, kynurenine, indole, and indoacetic and indoleproprionic acid derivatives [207,208,209,210,211]. These tryptophan metabolites modulate key physiologic activities, including the functioning of the immune system [212,213]. Elevated levels of kynurenine inhibit the immune response, consequently predisposing to cancer development [214,215]; conversely, indole and its derivatives suppress inflammation, thereby exerting a protective effect against cancer [216,217]. Additionally, tryptophan derivatives alter tumor cell behavior. Kynurenine stimulates cancer growth and tumor spread; indole metabolites induce tumor cell cycle arrest and cell death [218,219]. Finally, certain classes of indoles, the indolimines, as previously noted, exert genotoxic effects to induce nucleic acid damage and genomic instability. Knowledge of the interactions among the varied bioactive molecules of the gut microbiome and how these interactions affect cancer behavior and therapeutic response inform and optimize cancer care. The accession and structuring of such knowledge, however, creates a formidable analytic and computational challenge. One solution to this challenge may emerge from a powerful scientific discipline: systems biology.

### 4.4. Understanding the Microbiome from a Systems Biology Perspective

Complex biological phenomena result from interactions among, frequently, a dauntingly large number of molecular and physiologic variables; comprehensive assessment and integration of these interactions demands significant, possibly prohibitive, commitments of data processing expertise and scientific resources. Understanding these interactions yields critically important insights; identifying, describing, and structuring the summary effects of these interactions has significant scientific and clinical implications, potentially establishing pathways for the prediction of biological behavior and the creation of therapeutic drugs.

Systems biology, an integrative, computational discipline, paves these investigatory pathways through the construction of accessible mathematical models that emulate biological population assemblage, architecture, and performance [220,221,222]. These mathematical models provide a more efficient, incisive, and intuitive accounting of complex biological phenomena. Systems biology and the mathematical models they create help manage the extreme volume and disambiguate the complexity of gut microbiome interactions. To achieve efficient disambiguation, systems biologists utilize various highly versatile analytical methods, such as the production of genome-scale metabolic models (GEMs).

Genome-scale metabolic models (GEMs) computationally reproduce microbiome dynamic metabolic states through ensemble integration of “omic” (i.e., genomic, transcriptomic, proteomic, metabolomic) data [223,224,225]; ensemble models reliably predict organism behavior in response to external stimuli and anticipate downstream resultant effects [226,227]. GEMs comprehensively forecast the physiological interactions, integrated metabolic states, and reactions to environmental stressors (e.g., antibiotic use, toxin exposure, dietary fluctuation) of the gut microbiome [228,229,230]. Microbiome forecasting aids the preventative maintenance of eubiosis and therapeutic remediation of oncobiosis. Beyond leveraging metabolic-based forecasting, systems biologists may gain insights into the state of the microbiome through the assessment of structural and immunologic conditions [231,232].

Individuals exhibit unique microbiome structure defined by overall bacterial composition and biological interactions within the gut [233,234,235]. This structure derives from evolutionarily driven, specialized adaption to the gut biologic niche [236,237,238]. The microbiome structure within the niche affords both a gut-protective physical barrier as well as an optimized biochemical milieu; disruption of this structure risks invasion and colonization by intrusive and oncobiotic bacteria [239,240]. Comprehensive omics-derived profiling models provide keys to clarify the molecular basis of optimized microbiome compositional structure.

Alongside the emergence of an onco-protective compositional structure, the microbiome modulates immune function to defend against cancer initiation [241,242]. The gut microbiome conditions the immune system by training dendritic cells and T-cells through several molecular mechanisms, producing important antiviral substances, and beneficially regulating the activity of regulatory cells [20,243,244,245,246,247]. The scientist may understand these immunological effects through the lens of omics-based molecular profiling and systems biology modeling of microbiome–immunologic system interactions [248,249,250]. Genetic, genomic, and epigenetic constitution informs eventual composition and function of the gut microbiome; perturbations in composition and function negatively degrade salutary immune system operations, ultimately predisposing to neoplastic transformation and progression [251,252,253,254].

Systems biological approaches have transformed our insights into the molecular mechanics of the gut microbiome. These insights help preserve health and may prevent disease, especially cancer. Powerful analytic methods and expert investigation accelerate these insights. The continued adoption of pioneering computational and mathematical methods promises to advance system biological methods and hasten scientific discovery. New methods in artificial intelligence (AI) and their application to microbiome research afford inventive strategies to realize this promise more quickly.

### 4.5. Leveraging AI to Discover Gut Microbiome Structure and Function

AI plays an ever-burgeoning role in oncology, representing an important technique to abet cancer care [244,255,256]. For example, oncologists employ AI to optimize therapeutic decision-making, symptom management, and cancer diagnostic workflows [257,258,259]. AI applications work well to perform analyses of intimidatingly large data sets and discover solutions to complex, multifactorial problems; given the immensely complex nature of the gut microbiome, AI offers resolution towards more efficient and determinative elucidation of both healthy and pathological colonic microbiome processes. AI has the potential to improve description of the temporal and spatial composition, function, and therapeutic management of the gut microbiome.

#### 4.5.1. AI Insights into the Temporal/Spatial Structure of the Gut Microbiome

The gut hosts a vast array of bacteria, staggering both in absolute number and diversity of microorganisms present: various estimates place the quantities close to 100 trillion individual organisms comprising nearly one thousand different bacterial species [260,261]. Moreover, gut microbiome composition changes dynamically, continually altering in response to a variety of factors, including age, disease, medications, and environmental exposures. Adding to quantitative complexity, the colon exhibits a spatial diversity that depends, in part, upon different gut geographic, physiologic, and structural conditions. Scientists increasingly harness the power of AI-based approaches to assemble, process, and analyze information from the massive data stores generated from microbiome studies [262,263]. This information provides valuable insight into the temporal, spatial, and operational structure of the gut microbiome and, ultimately, the development and implementation of therapeutic cancer strategies.

#### 4.5.2. AI Methods, Models, and Algorithms to Investigate the Function and Operations of the Gut Microbiome

The gut microbiome executes several integral biochemical, metabolic, and physiologic functions. From these functions emerges an intricate and adaptive biological system. The analytic conceptualization of operational relationships among the enormous number of microbiome organisms routinely challenges the capabilities of traditional computational methods. To overcome these challenges, microbiome scientists have available to them a variety of AI methods, models, and algorithms.

Machine learning, a pivotal AI method, enhances the efficiency, rigor, and speed of gut microbiome analyses. Machine learning enables computer systems to process data autonomously, iteratively improving the quality and precision of analytic outputs through serial, expanding data exposition learning cycles. Scientists have employed machine learning methods to identify important gut microbiome molecular signatures. Machine learning methods have advanced gut microbiome data preprocessing [264]. Computational biologists have leveraged machine learning methods to better understand the molecular underpinnings of gut microbiome network interactions. The application of highly effective machine learning methods and the utilization of innovative mathematical models and statistical algorithms allow computational biologists to unveil the intricate functioning of and interactions within the gut microbiome.

Machine learning methods segregate into two broad categories: supervised and unsupervised. Supervised machine learning relies on and employs input data with previously established output values. Investigators have employed gut microbiome-supervised machine learning methods to improve cancer clinical diagnosis [265], prognosis [266], and treatment [267].

In contrast to supervised methods, unsupervised machine learning does not rely upon input data sets with known output values or associations; rather, unsupervised machine learning seeks to discover patterns, commonalities, and disparities among input data elements. These discovered groupings allow biologists and clinicians to define and understand organizational and functional relationships among constituents of the gut microbiome. Unsupervised machine learning methods have provided insight into the population characteristics of distinct microbiome gut microbiome consortia [268], identify previously unknown gut microbiome biomarkers [264], and distinguish patient populations based on their gut microbiome profiles [269]. Supervised and unsupervised methods rely upon a repertoire of key mathematical models and statistical algorithms.

Primary supervised machine learning models and algorithms include logistic regression, support vector machines, and random forests. Both logistic regression and support vector machines allow for the binary classification of output results. The random forest algorithm also solves classification problems but arguably, compared with other supervised approaches, performs more optimally and efficiently with data sets having inherently complex relational structures. These supervised machine learning models and algorithms have supported gut microbiome research examining disease prediction [270], differences between healthy and dysfunctional microbiome states [271], and gut microbiome functional capacities [272].

Unsupervised models and algorithms aid the discovery and elucidation of unknown structures and interactions among data elements. Computational biologists and AI engineers frequently employ three models and algorithms to perform unsupervised machine learning tasks: K-means clustering, hierarchical clustering, and principal component analysis.

K-means and hierarchical clustering categorize input data elements based on maximizing shared characteristics within a class and distinctions among classes. These clustering approaches, though, employ different clustering strategies and individually better suit specific applications. K-means clustering creates multiple independent clusters, whereas hierarchical clustering creates progressively more granular dendritic clusters. Vis-à-vis K-means clustering, hierarchical clustering offers a more intuitive insight into logical relationships among clusters. Hierarchical clustering expends more computational resources relative to K-means clustering, ideally suiting hierarchical clustering for the analysis of smaller data sets.

Principal component analysis aids and accelerates unsupervised machine learning by reducing large, intractable data sets to less complex, more manageable sets by employing only the most relevant (i.e., principle) variables within a data set or by compressing the data set into fewer, more information-dense, synthetic variables. These models and algorithms have enabled the identification of gut microbiome enterotypes [273], elucidation of pathogenic microbiome conditions [274], and summary descriptions of gut microbiome populations resulting from diet and body habitus [275].

Together, these metagenomic, metabolomic, systems biology, and computational approaches have led to a deeper understanding of gut microbiome genomic structure and functioning. This deep understanding provides a foundation for the development and implementation of therapeutic inventions to maintain eubiosis and ameliorate dysbiosis [276,277,278].

## 5. Gut Microbiome Therapeutic Intervention

Three foundational, transformative approaches drive clinically meaningful intervention of the gut microbiome: genetic engineering/synthetic biology, prebiotic/probiotic implementation, and fecal transplantation.

### 5.1. Genetic Engineering and Synthetic Biology

#### 5.1.1. Genetic Engineering

Genetic engineers directly edit the genome of organisms to achieve desired phenotypic outcomes; gut microbiome scientists engineer bacterial genomes to promote the growth of beneficial bacterial species, enhance the synthesis of clinically advantageous metabolic products, and attenuate the production of harmful byproducts [279,280,281]. For example, clinicians have leveraged genetic engineering technologies to improve therapeutic outcomes and minimize untoward side effects; investigators have used genetically-modified Bacillus coagulans bacterial spores to release nanoparticle-bound chemotherapeutic agents in the intestine, thereby improving focused drug delivery to sites of active colorectal cancer [282]. More recently, two specific genetic engineering advances, base editing and in situ conjugation, have facilitated the translation of gut microbiome engineering into the clinic.

Molecular biologists developed base editing, a modified form of CRISPR-Cas9 gene editing, to ensure greater precision [283,284]. With standard CRISPR-Cas9 gene editing, a complementary RNA guides the endonuclease enzyme Cas9 to a specific target nucleotide sequence within the genome. Cas9 then excises a multi-nucleotide section of the genome that flanks the target sequence; subsequently, the cell uses homology-directed repair (HDR) employing a template containing an altered nucleotide sequence to replace the target sequence. With this standard approach, because it involves the excision of multiple nucleotides and relies upon imperfect HDR to achieve repair and editing, errors may occur. In comparison, the base editing technique utilizes a variant Cas9 that allows accurate localization to a target nucleotide sequence but does not perform nucleotide excision; instead, base editing employs a deaminase to alter the targeted nucleotide sequence [285,286,287]. Base editing ensures more proficient and precise gene editing. Scientists recently demonstrated successful editing of the gut microbiome using base editing together with a highly effective phage-based payload delivery system to achieve large-scale, in situ gene editing [288].

Bolstering clinicians’ capabilities to effectively modify the gut microbiome, investigators have adapted another molecular delivery approach to facilitate high efficiency in situ gene editing. This approach, **M**etagenomic **A**lteration of **G**ut microbiome by **I**n situ **C**onjugation (MAGIC), achieves wholesale gene editing of an entire microbial community in the living organism [289,290]. MAGIC utilizes the bacterial gene transfer molecular mechanism of conjugation to introduce, in vivo, a variant gene sequence from a donor bacterium into a bacterially diverse recipient community. MAGIC permits genetic engineering of the entire bacterial communal ecosystem while at the same time preserving the inherent, intricate, and frequently unstable state of interactions among heterogenous bacteria. In an inaugural series of experiments, investigators demonstrated the ability to simultaneously genetically engineer nearly 300 significantly varied gut bacterial species. Genetic engineering methods and technologies afford means to not merely modify existing genes but, more broadly, to introduce new genetic capabilities into the microbiome; another technological advance, synthetic biology, applies the insights of genetic engineering to accomplish practical implementation of these new capabilities.

#### 5.1.2. Synthetic Biology

Synthetic biology capitalizes on multiple scientific disciplines to aid in the design and rendering of novel, artificial biologic systems; these systems derive from systems biology-based computational conceptualization, molecular biological modification, and clinical implementation of customized microbiome consortia [291,292,293]. Recent inventions and discoveries, such as the creation of environmental gut biosensors, the construction of synthetic bacterial consortia, and the manufacturing of engineered metabolic pathways, demonstrate the potential of synthetic biology.

Biologists have modified gut bacteria, including Escherichia coli, Bacteroides, and Lactobacillus, to carry molecular payloads that function as biosensors capable of detecting and reporting conditions of dysbiosis and harmful metabolite production [294,295,296]. These gut biosensors demonstrate abilities to identify numerous small molecules, notably, inflammatory byproducts, GABA metabolites, and SCFA derivatives [297,298,299,300]. The engineered, biosensing bacteria reliably integrate into the native gut microbiome ecosystem, creating a novel, stable, self-reporting gut bacterial ecosystem.

Gut scientists fabricate synthetic gut bacterial consortia comprising bacterial populations able to thrive within the complex gut environment and selected or genetically engineered to mitigate cancer risk, improve therapeutic outcomes, or enhance clinical research. Investigators report the successful development and utilization of several synthetic microbial consortia, for example, to treat colitis [301], improve precision therapeutic responses [302], and facilitate experimental modeling of the gut microbiome [303]. Additionally, synthetic biology furnishes researchers with the technical abilities to fashion and assemble innovative gut bacterial systems to promote microbiome investigation. As a case in point, scientists constructed an optimized bacterial population comprising 100 gut bacterial species to emulate the functioning of a well-performing, elderly gut microbiome [304]—the MCC100 system. MCC100 represents a synthetic bacterial system that mimics that of older individuals and affords a theoretical opportunity to study the health consequences of and therapeutic drug responses influenced by the elderly gut microbiome.

Investigators introduced another synthetic microbial consortium recreating a microbiome that generates butyrate metabolites; this consortium furnishes a dedicated platform for focused investigation of this butyrate’s biological and health effects [305]. Synthetic biology undergirds other gut metabolic models, such as those with active bacterial propionate and acetate biochemical production pathways [306,307,308,309]. One research study documented the development of a synthetic propionate-producing microbiome consortium capable of restoring compromised mitochondrial function [310]. Other studies leveraged principles of synthetic biology to design, formulate, and assemble gut microbial populations optimized for the biomanufacturing of acetate [311,312]; such acetate-generating gut bacteria improve gut function and overall health [313,314]. To further improve clinical outcomes, scientists and clinicians have available to them additional options to accomplish the directed modification of the gut microbiome: prebiotic and probiotic administration and fecal transplantation.

### 5.2. Advances in Gut Microbiome PreBiotics, ProBiotics and Fecal Transplantation

Prebiotics, probiotics, and fecal transplantation alter the compositional architecture of the microbiome. Prebiotics supply substrates and nutrients that induce preferential growth of selected bacteria [315,316,317]. By supporting the growth and proliferation of specific bacteria, prebiotics significantly influence microbiome constitution, function, and metabolite generation. For instance, high-fiber prebiotics foster the expansion of healthy, salutary bacteria by conferring a growth advantage over damaging, toxic species [318]. Prebiotics rich in fructose, galactose, and xylose oligosaccharides stimulate the growth of bacteria that boost the function of the immune system [319]. Complex carbohydrates, frequently a component of many prebiotics, sustain bacteria that produce beneficial SCFAs, specifically acetate, propionate, and butyrate [320]. Inulin-containing prebiotics promote the growth of beneficial gut microbiota, such as Bifidobacterium, Enterococcus faecalis, and Lactobacillus [321,322,323]. Several clinical trials have demonstrated favorable effects of prebiotic inulin supplementation in patients undergoing active cancer treatments. Investigators have established that inulin supplementation effectively reduces systolic blood pressure in women undergoing neoadjuvant chemotherapy for early-stage breast cancer [324]. In colon cancer patients, the use of inulin prebiotics results in increases in immune system-boosting interferon-gamma [325]. In a randomized, double-blind, placebo-controlled trial, radiation therapy-treated gynecologic cancer patients demonstrated a quicker recovery of beneficial gut microbiome composition and stool quality after receiving a prebiotic inulin-containing dietary supplement [326]. Attaining the benefits of prebiotics, though, necessitates chronic prebiotic administration; furthermore, the realization of such benefits may significantly lag the initiation of prebiotic use. In contrast, patients may experience more immediate and potentially durable benefits with probiotic use.

Probiotics contain custom-formulated, live, active bacterial mixtures; vis-à-vis prebiotics, probiotics act more directly and determinatively to transform microbiome composition [327,328,329]. The creation of probiotic formulations relies on several compounding strategies, including the laboratory amalgamation of multiple beneficial bacterial cultures [330,331], genetic bioengineering of preexisting bacterial communities to enhance selected biological properties [332,333,334], and the ex vivo development of novel bacterial communities to create artificial, health-enhancing bacterial ecosystems [335,336]. Clinicians administer probiotics in various forms—particles, emulsions, and capsules, among others—to optimize transit through and survival within the gut [337,338,339]. Probiotics may prevent cancer [340,341], improve therapeutic responses [342,343], and ameliorate drug-associated side effects [344,345]. Results from multiple clinical trials have reinforced the utility of probiotics as a therapeutic adjunct. In colorectal cancer patients, in the postoperative period, the consumption of Lactobacillus and Bifidobacteria probiotic formulations reduced pro-inflammatory cytokine levels to optimize the immune state of the recovering colon [346]. The concurrent use of a probiotic cocktail in a Phase II trial of patients receiving radiotherapy for the treatment of nasopharyngeal carcinoma reduced mucositis incidence rates [347]. In a phase I trial, the use of a Clostridium butyricum probiotic strain (CBM588) in metastatic renal cell carcinoma patients receiving immune checkpoint therapy prolonged progression-free survival and response rates [348]. An alternative approach to modify directly the gut microbiome, fecal transplantation, may demonstrate advantages over probiotic use [349,350,351]. Fecal transplantation directly introduces an optimized fecal bacterial combination into the gastrointestinal system, utilizing proximal or distal routes of transfer [352,353].

Fecal transplantation administers a carefully selected fecal bacterial sample proximally, employing nasogastric instillation or capsule ingestion, or distally, using colonoscopic or enema seeding [354,355,356,357,358,359]. Clinicians obtain fecal samples for transplantation from healthy patient donors who fulfill specific medical criteria that include good general and, specifically, gastrointestinal health, no recent antibiotic use, the absence of communicable disease, no history of high-risk behaviors such as intravenous drug use or sexual activity with infectious potential, and no recent travels to areas with elevated rates of infectious disease [360,361]. Fecal transplantation, as with prebiotics and probiotics, may reduce cancer risk [362,363], mitigate drug side effects [364], and improve clinical outcomes in patients receiving immunotherapy and other cancer treatments [365,366,367,368,369]. Moreover, fecal transplantation may achieve therapeutic endpoints more quickly and effectively compared with other gut microbiome interventions [370,371,372,373,374]. The recent Federal Drug Administration (FDA) approval of two fecal transplant therapies underscores the increasing clinical currency of this treatment approach. Based on multiple randomized, placebo-controlled clinical trials [375,376], the FDA, in 2022, approved enema administration of the live fecal microbiota suspension RBX2660 (Rebyota) to prevent the recurrence of gastrointestinal tract Clostridioides difficile infection (CDI) following treatment for recurrent CDI. Clinicians observed that a single administration of RBX2660 safely and durably prevented recurrence; moreover, RBX2660 demonstrated superior clinical efficacy compared with standard-of-care treatment. More recently, in 2023, the FDA also granted approval for SER-109 (Vowst), an orally administered live fecal microbiota spore formulation, for the prevention of CDI recurrence following antibiotic treatment [377]. In a randomized, placebo-controlled trial assessment, SER-109 therapy resulted in less frequent CDI recurrence compared with the placebo controls [378]. As an orally administered agent, SER-109 represents an effective, less invasive, and more convenient therapy compared with gastrointestinal tract instillation or enema dispensation of a live fecal agent [379].

## 6. Pathways to Accelerating Microbiome-Related Discovery and Clinical Translation

As investigators and clinicians continue to increasingly recognize the importance of microbiome function as a determinative factor in cancer outcomes, considerations emerge as to how to best accelerate microbiome-related scientific discovery and clinical translation. Such discovery and translation, though, may create resource, logistical, and practical issues [380,381,382]. Limitations in computational expertise, laboratory technologies, and patient access may curtail advancements of our knowledge of the microbiome. Team collaborative investigations afford multidisciplinary capabilities to overcome these limitations and elevate the science of microbiome research and therapeutic innovation. The following sections examine successful microbiome team initiatives with a specific emphasis on the unique team academic research and clinical environment at the City of Hope Comprehensive Cancer Center.

### 6.1. Team Collaborative Science Accelerates Gut Microbiome Research

Investigators now rely increasingly on team science to achieve research goals. Team science allows investigators to pool resources and more easily access experts, patients, and research samples. Team-focused initiatives reduce the costs of and accelerate research, resulting in improvements in the quantity and quality of research. Team science may comprise collaborations among different departments within a single institution, different institutions within a nation, or multiple nations across the globe. Examples of successful microbiome team initiatives include the National Institutes of Health’s Human Microbiome project (HMP), the Tri-Service Microbiome Consortium (TSMC), and the Global Microbiome Conservancy (GMC).

The HMP, a ten-year initiative supported by the National Institutes of Health, sought to gain deeper insight into the human microbiome. The HMP promoted collaborative partnerships among Virginia Commonwealth University, Washington University, the Baylor College of Medicine, and several other academic institutions to investigate the microbiome. Phase One (2007–2014) of the HMP involved creating preliminary descriptive genomic data sets, developing investigatory tools, and establishing regulatory guidelines to facilitate microbiome research. In Phase Two (2014–2016), designated the Integrated Human Microbiome project, the team aimed to uncover causal relationships between microbiome conditions and states of health and disease. Among its many high-impact contributions to advancing microbiome research, the HMP presented one of the first comprehensive structural descriptions [234], detailed the metabolic functioning [383], and identified major causative microbial pathogens [384] of the microbiome. The HMP laid the early foundation from which other microbiome research collaborations arose.

The Department of Defense chartered the TSMC in 2016 “to enhance collaboration, coordination, and communication of microbiome research” [385]. The TSMC includes personnel from the Army, Navy, and Air Force; academic faculty and students; and commercial industries. Recent efforts of the TSMC have helped to characterize the human microbiome, advance computational and research methods, and create novel in vitro and in vivo experimental models. The TSMC places great emphasis on leveraging the potential of team microbiome investigation to maintain levels of health and optimal human functioning in changing and stressful environmental conditions. Both the HMP and TSMC highlight the value of domestic, primarily local and national, collaboration; international team microbiome research has also yielded valuable scientific discoveries to advance our understanding of the microbiome.

The GMC, established in 2016 by microbiologists at the Massachusetts Institute of Technology, seeks to expand the understanding of the microbiome, increase related research volume, and serve as a repository and source of globally diverse microbiome samples [386]. The GMC has collected specimens from around the world, including Africa, the Arctic, North and South America, Europe, and Asia [387,388,389]. Access to and utilization of this collection enables the discovery of global demographic, economic, and societal effects on microbiome composition and functioning that impact human health and well-being. Work employing samples from the GMC has described the molecular consequences of industrialization on microbiome genetic diversity [390,391] and explored the worldwide distribution and prevalence of microbiome epigenetic modulation [392] and the consequences of urbanization on microbiome population dynamics [393]. The domestic and global collaborative efforts of the HMP, TSMC, and GMC help to expand the breadth of microbiome research; institutional, intramural efforts promise to redouble the depth of research. Underscoring this promise, the unique academic research and clinical environment at the COH Comprehensive Center demonstrates the tremendous potential of intramural team efforts at an academic cancer center to force multiply the impact of collaborative microbiome research.

### 6.2. The COH Model of Microbiome Collaborative Team Science

#### 6.2.1. The Academic/Community Oncology Alliance at the COH

Emerging economic, governmental, and societal trends have driven the establishment and growth of the alliance between community and academic oncology practices [394]. The COH Comprehensive Cancer Center illustrates the power and potential of this alliance. The unique structure of COH, with its centralized academic Duarte, California hub, and expansive network of community practice sites, allowed for the development of an exceptional, mutually beneficial clinical and investigational synergism [395,396,397].

#### 6.2.2. The COH Clinical Network

**Figure 3:**
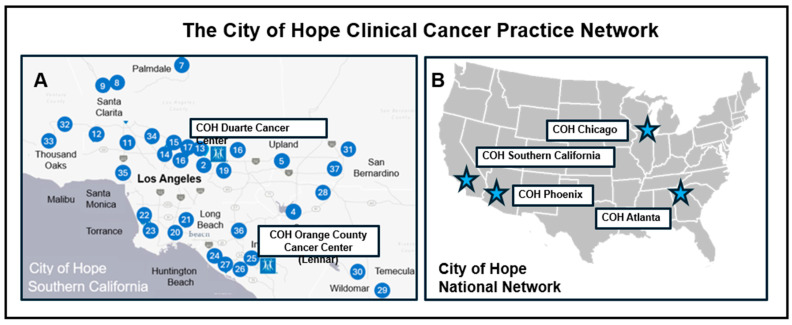
**COH operates a national cancer treatment network.** (**A**) COH provides care to patients in southern California through a network of more than thirty community oncology sites and an academic oncology hub in Duarte, California. (**B**) COH has a national presence with operations in Phoenix, Arizona; Atlanta, Georgia; and Chicago, Illinois, in addition to its community and academic practices in southern California.

COH provides cancer care across the nation with operations in the Southwestern, Midwestern, and Eastern regions of the United States, with outpatient facilities located in the Chicago, Atlanta, Phoenix, and Southern California areas. These operations serve more than 100,000 new patients each year; over 500 physicians and 10,000 support team members provide this care [398]. COH concentrates care in Southern California, with care readily available to the nearly twenty million inhabitants of the Los Angeles, Orange, Riverside, and San Bernardino counties. The Southern California Duarte campus functions as a central organizational hub for more than thirty satellite community oncology practices. A second, recently constructed subsidiary COH Cancer Center (Lennar) in Orange County, California, has operated since 2022. Together, the COH academic and community oncology enterprise delivers services to a broad socio-economically and racially diverse patient population (Figure 3). This large and diverse clinical network helps sustain a robust and innovative microbiome research operation at COH.

**Figure 4:**
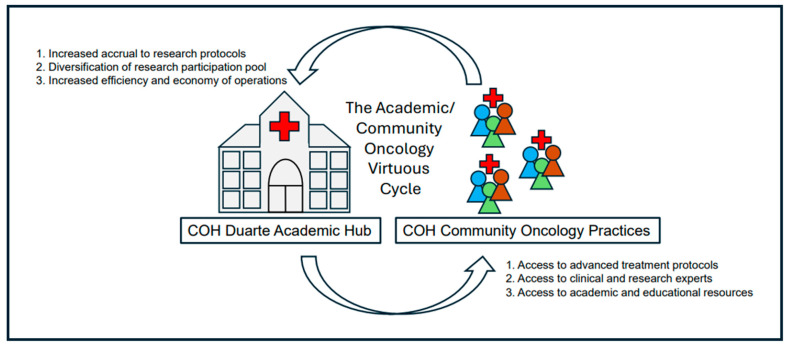
Academic and community oncology practices create a virtuous cycle of increased clinical research and improved patient care. The integration of academic and community oncology care at COH results in reciprocally beneficial research and optimized patient treatments.

The Duarte hub provides all providers and patients access to advanced, innovative therapeutic treatments and educational resources, the ability to interface with world experts in clinical care and experimental methods, and the opportunity to improve the diversity and relevance of research discovery through the involvement of their community patients in investigational protocols. Conversely, the COH academic research hub benefits from the participation of community oncology practices by increasing the overall number and diversity of subjects in their studies. Increased numbers help studies to more quickly reach critical accrual numbers and reduce population bias, thus minimizing the potential for statistical skew and contributing to a logistical economy of scale in protocol completion. The mutually beneficial synergism between COH community oncology practices and the Duarte academic hub has led to a virtuous cycle of enhanced research productivity and health care; this virtuous cycle extends to microbiome-focused investigation and clinical translation (Figure 4).

#### 6.2.3. COH Resources Available for Microbiome Research

**Figure 5:**
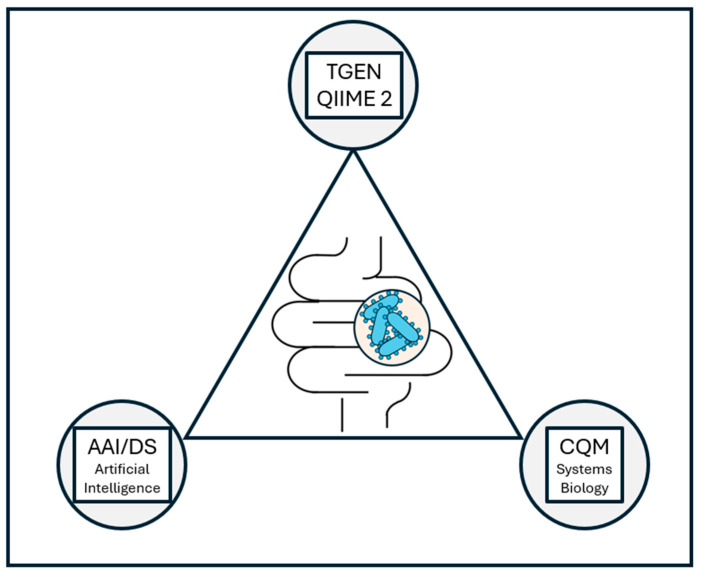
**City of Hope has numerous resources available to facilitate microbiome research.** To its network enterprise COH makes available a variety of experimental and computational resources to accelerate microbiome research.

COH makes available a variety of resources to clinicians and investigators to assist with microbiome-related research. COH situates its research resources centrally at the Duarte academic campus and as stand-alone facilities administratively closely linked with the Duarte hub. Core facilities work in close collaboration with investigators to assist with the design, execution, and data analysis of research investigations. The COH-associated Translational Genomics Research Institute (TGen), with its Integrated Microbiome Center (IMC) and its work with the Quantitative Insights Into Microbial Ecology (QIIME 2) project, the COH Computational and Quantitative Medicine Department (CQM), and the COH Department of Applied Artificial Intelligence and Data Science (AAI/DS) have focused their efforts on advancing microbiome research (Figure 5).

## 7. TGen, IMC, and QIIME 2

### 7.1. TGen

TGen, a genomics research institute that has been affiliated with COH since 2016, aspires to make precision medicine a reality for all. TGen has physical locations at two campuses located in Arizona connected with the COH Duarte academic hub through a high-capacity, ultra-high-speed data transmission network. TGen develops and implements new methods of molecular genetic assessment with the aim of optimizing cancer diagnostics, prognostics, and personalized, targeted cancer therapeutics. TGen researchers and data analytic teams work with clinicians, translational investigators, and basic scientists throughout the COH network to advance the frontiers of genomic science. TGen plays an integral role in microbiome research at COH, primarily through the operation and administration of the IMC.

### 7.2. IMC

The TGen IMC functions as a multidisciplinary core research service that facilitates the efforts of COH oncologists and scientific investigators to understand and leverage the microbiome in cancer care. The IMC offers streamlined assistance for microbiome-related studies. Assistance includes study design consultation, specimen collection protocols, nucleic acid extraction, sequencing library preparation, next-generation sequencing, post-sequencing computational and statistical analyses, and downstream translational interpretation. Importantly, the IMC adheres to Clinical Laboratory Improvement Amendment (CLIA) standards, ensuring the reliability and security of microbiome analyses. IMC computational analyses rely heavily on the QIIME 2 bioinformatics platform.

### 7.3. QIIME 2

QIIME 2 evolved from QIIME, a data science suite for the high throughput computational analysis of microbiome 16S and 18S rRNA amplicon sequencing data [399,400]. Developers reengineered QIIME to accommodate next-generation microbiome computational processing, including the assessment of microbiome-related metabolomic and metagenomic data. The reengineered version, QIIME 2, allows for dynamic, organic software evolution through interactive community application development [401]. QIIME 2 provides transparent, reproducible data-provenance tracking with archival storage of all analytic steps. Currently, the QIIME 2 development team operates as an integral element of the COH IMC involved with core computational processing of microbiome data. 

## 8. The COH CQM

The COH CQM advances multidisciplinary projects in computational biology to improve cancer diagnostics and therapeutics. CQM comprises several core divisions: Computational Structural Biology and Drug Design, Biostatistics, Informatics, Mathematics for Cancer Evolution and Early Detection, Health Analytics, and a Division of Mathematical Oncology and Computational Systems Biology. All CQM divisions contribute to the advancement of microbiome-related research at the COH. Of note, Computational Systems Biology at the COH focuses on developing models describe complex biological interactions, an approach particularly well suited to understanding the structure and function of the gut microbiome. The Computational Systems Biology Division works to develop collaborative relationships with COH clinicians, basic scientists, and translational oncologists.

## 9. COH AAI/DS

AI continues to transform clinical medicine. COH created the AAI/DS to accelerate independent AI investigation; establish a resource for AI instruction, discussion, and education; and foster collaborative AI-based research projects. AAI/DS faculty and staff assist with experimental design, execution, and data analyses. In association with COH investigators, AAI/DS has developed several machine-learning-based models to accurately mirror and understand actual clinical scenarios. The AAI/DS introduced models to better elucidate the structure and function of the gut microbiome; one machine learning application analyzes the composition of the gut microbiome to predict the risk of adverse gastrointestinal side effects during breast cancer treatment [402].

## 10. Microbiome Advances at COH

COH clinicians and scientists have made significant insights into the role of the microbiome in clinical oncology and continue active engagement in high-impact, innovative microbiome research. COH investigators recently published several high-impact gut microbiome-related studies, including investigations examining the response to cytotoxic, molecularly targeted, radiation, and CAR-T cell therapies [403,404,405,406,407,408]; other important COH gut microbiome studies have focused on ameliorating cancer-related cachexia [409], sarcopenia [410], graft versus host lethality [411], long-term cancer mortality [412], and cancer risk [413]. COH researchers currently oversee multiple federally funded research projects interrogating the role of the gut microbiome in graft versus host disease, metabolic surgery, response to cancer immunotherapy, gut–immune system interactions, and intestinal genomic diversity [414]. With the aim of advancing our understanding of the clinical importance of the gut microbiome in cancer care, clinical trials at the COH seek to evaluate microbiome-related clinical outcomes, therapeutic responses, and prevention of toxic side effects. (Table 1). Building on these initiatives, COH continues to accelerate the pace of microbiome discovery through expansion of its research and computational resources.

## 11. Meeting the Challenges of Gut Microbiome Research at COH

As gut microbiome research continues to grow, challenges arise, potentially impeding its progress. These challenges arise due to the novelty and unfamiliarity but also the inherent complexity and sheer breadth of gut microbiome investigation. Among numerous challenges, three have acquired recent prominence: funding limitations, standardization of methods, and ethical concerns.

Investigators face fierce competition for research funding. Limited resources and competing demands drive this competition. In the case of gut microbiome research, additional considerations further hamper investigators’ competition for financial support. Conceptually, modification of the gut microbiome remains incompletely proven as an effective clinical strategy; as such, granting agencies may preferentially fund those interventions that have more firmly demonstrated clinical promise. Further, the daunting complexity of the gut microbiome may not only impede research progress but also mute enthusiasm for funding as agencies hesitate to fund what they do not understand. Finally, the dearth of established experts and competent research facilities may discourage funding bodies as they deem gut microbiome research both impractical and wasteful.

The standardization of gut microbiome methods also presents challenges to the advancement of research. As a novel investigation discipline, gut microbiome researchers continue active refinement of experimental methods; with such refinement comes the ongoing adoption of new experimental and clinical protocols. Such experimental flux leads to disparities among research approaches and results, potentially invalidating cross-platform comparisons. This issue becomes particularly acute when investigators conduct clinical trials as they often seek to compare their results with those of previous trials. Moreover, the lack of stable, standardized gut microbiome methods may compel investigators to continually revise research protocols—a potentially cost-prohibitive and time-consuming proposition.

Ethical issues, specifically, considerations of informed consent, beneficence, and privacy, also present challenges to microbiome research. Informed consent obligates the investigator to provide an accurate, comprehensive explanation of the proposed experimental intervention; yet, given the complexity and rapidly evolving nature of microbiome experimental methods and protocols, whether investigators can achieve adequate explanations raises ethical concerns. The ethical principle of beneficence directs physicians to advance patients’ interests. Our understanding of the harms, risks, and benefits associated with gut microbiome interventions, though, continues to mature; without a reasonably complete accounting of the side effects associated with the manipulation of the microbiome, the valid achievement of beneficence remains elusive. Finally, concerns for patient privacy arise with gut microbiome research. Gut microbiome research frequently generates copious volumes of patient-specific genomic data; these data potentially link specific patients with serious health risks and active disease states. The completion of research studies may require the publication of all data, including complete genomic sequencing information that potentially reveals patient identity and jeopardizes privacy.

Funding limitations, methods standardization, and ethical concerns threaten the progress of gut microbiome research. The resolution of these challenges promises to accelerate research discovery; the COH academic cancer network model offers a pathway to achieve efficient resolution. The microbiome support services provided through QIIME 2 and other academic departments at COH provide investigators with the most advanced and contemporary tools to assist with the design and execution of microbiome experiments and clinical trials. These tools help ensure not only the highest quality microbiome research but also optimally position investigators for funding success. QIIME 2 has built a reputation as a pioneer in the development of microbiome experimental and computational platforms; furthermore, QIIME 2 makes these platforms openly available to the worldwide microbiome community. Such wide-scale availability promotes the standardization and adoption of universal microbiome research protocols. Lastly, the COH sponsors an expansive portfolio of clinical trials. The COH Institutional Review Board and Clinical Trials Office oversees the ethical and administrative conduct of all trials, including microbiome-focused protocols. Microbiome clinical research adheres to the same strict oversight regulations as other clinical trials at the COH; this adherence helps guarantee maximal patient safety and the protection of personal privacy rights. The COH model of integrated interdepartmental cooperativity illustrates the powerful potential of operational synergism to overcome the emerging challenges associated with gut microbiome research.

## 12. Summary and Conclusions

Clinicians and scientists increasingly recognize the importance of the microbiome as a guardian of health and defender against cancer. The optimal constitution and function of the colonic microbiome produces eubiosis; disruption of eubiosis leads to dysbiosis and possible oncobiosis and initiation of cancer. The emergence of omic-based approaches in medicine and biology permits a granular understanding of the constitution of the microbiome. Reversing dysbiosis and oncobiosis, we observe, requires a functional understanding of the microbiome.

The quantitative and relational complexity of the microbiome strains our ability to achieve functional insight. Innovative biological and analytic approaches provide workable pathways to increase our understanding of molecular function and devise therapeutic interventions. System biology approaches permit tractable means of understanding functional relationships among vast arrays of bacterial data. Artificial intelligence allows us to develop an understanding of interactions among immensely complex microbiome events. The practical utilization of systems biology and artificial intelligence requires access to expertise and investigatory resources.

The community oncology and academic cancer center network alliance provides a practical model to access and maximally leverage needed expertise and resources. Community oncology practices provide access to a wide diversity and large volume of patients; academic cancer centers make available specialized expertise and investigational and scientific resources. Together, community oncology practices and academic cancer centers synergize to accelerate microbiome discovery. The COH national cancer center network illustrates the potential of team synergism. COH possesses the requisite elements—patients, academic resources, and an integrated cancer treatment network—to maximally advance microbiome-based research and therapeutic development.

## Figures and Tables

**Table 1:** Microbiome clinical trials at City of Hope.

Clinical Trial	NCT Identifier	Status
Study to Detect Changes in Urinary and Gut Microbiome During Androgen Deprivation Therapy and Radiation Therapy in Patients with Prostate Cancer	NCT04775355	Recruiting
CBM588 in Combination with Nivolumab and Cabozantinib for the Treatment of Advanced or Metastatic Kidney Cancer	NCT05122546	Active, not recruiting
TAC/MTX vs. TAC/MMF/PTCY for Prevention of Graft-versus-Host Disease and Microbiome and Immune Reconstitution Study (BMT CTN 1703/1801)	NCT03959241	Completed
A Multiple Dose Study to Evaluate Safety, Tolerability, PK, and Efficacy of SER-155 in Adults Undergoing HSCT	NCT04995653	Active, not recruiting
CBM588 Capsules in Combination with Nivolumab and Ipilimumab for the Treatment of Advanced Stage Kidney Cancer	NCT06399419	Recruiting
Adding Itacitinib to Cyclophosphamide and Tacrolimus for the Prevention of Graft Versus Host Disease in Patients Undergoing Hematopoietic Stem Cell Transplants	NCT05364762	Recruiting
CBM588 in Improving Clinical Outcomes in Patients Who Have Undergone Donor Hematopoietic Stem Cell Transplant	NCT03922035	Active, not recruiting
Minnelide and Osimertinib for the Treatment of Advanced EGFR Mutated Non-Small Cell Lung Cancer	NCT05166616	Recruiting
Atezolizumab, Guadecitabine, and CDX-1401 Vaccine in Treating Patients with Recurrent Ovarian, Fallopian Tube, or Primary Peritoneal Cancer	NCT03206047	Active, not recruiting
Probiotic Yogurt Supplement in Reducing Diarrhea in Patients with Metastatic Kidney Cancer Being Treated With Vascular Endothelial Growth Factor-Tyrosine Kinase Inhibitor	NCT02944617	Completed
CBM588, Nivolumab, and Ipilimumab in Treating Patients with Stage IV or Advanced Kidney Cancer	NCT03829111	Completed
Pembrolizumab, Endocrine Therapy, and Palbociclib in Treating Postmenopausal Patients with Newly Diagnosed Metastatic Stage IV Estrogen Receptor Positive Breast Cancer	NCT02778685	Recruiting
Study of IL-22 IgG2-Fc (F-652) for Subjects with Grade II–IV Lower GI aGVHD	NCT02406651	Completed
